# Four year follow-up of a randomised controlled trial comparing open and laparoscopic Nissen fundoplication in children

**DOI:** 10.1136/archdischild-2013-304279

**Published:** 2014-02-14

**Authors:** Maurizio Pacilli, Simon Eaton, Merrill McHoney, Edward M Kiely, David P Drake, Joseph I Curry, Keith J Lindley, Agostino Pierro

**Affiliations:** 1 Department of Surgery, Institute of Child Health and Great Ormond Street Hospital, University College London, London, UK; 2 Gastroenterology Units, Institute of Child Health and Great Ormond Street Hospital, University College London, London, UK

**Keywords:** Paediatric Surgery, Gastroenterology, Procedures

## Abstract

**Objective:**

To evaluate the 4-year results following a randomised controlled trial (RCT) comparing open (ONF) and laparoscopic (LNF) Nissen fundoplication in children.

**Background:**

It is assumed that long-term results of ONF and LNF are comparable. No randomised studies have been performed in children.

**Methods:**

A follow-up study was performed in children randomised to ONF or LNF (*clinicaltrials.gov* identifier NCT00259961). Recurrent gastro-oesophageal reflux (GER) was documented by upper gastrointestinal contrast study and/or 24-h pH study. Nutritional status, retching and other symptoms were investigated. A questionnaire was used to assess the quality of life before and after surgery.

**Results:**

Thirty-nine children were randomised to ONF (n=20) or LNF (n=19). There were 15 ONF and 16 LNF neurologically impaired children. One patient (ONF group) was lost to follow-up. Follow-up was 4.1 years (3.1–5.3) for ONF group and 4.1 years (2.6–5.1) for LNF group (p=0.9). Seven neurologically impaired children had died by the time of follow-up (3 ONF, 4 LNF). Incidence of recurrent GER was 12.5% in the ONF and 20% in the LNF (p=ns). One patient in each group underwent redo-Nissen fundoplication. Nutritional status improved in both groups, as indicated by a significant increase in weight Z-score (p<0.01). Gas bloat and dumping syndrome were present in both groups (p=ns). Incidence of retching was lower in the laparoscopic group (p=0.01). Quality of life improved in both groups (p=ns).

**Conclusions:**

Open and laparoscopic Nissen provide similar control of reflux and quality of life at follow-up. LNF is associated with reduced incidence of retching persisting at 4-year follow-up.

**Trial registration number:**

NCT00259961.

**What is known about this topic**

- The open Nissen fundoplication is an invasive surgical procedure associated with frequent postoperative complications and recurrence of reflux up to 30%. The laparoscopic Nissen fundoplication offers the advantage of decreasing the surgical trauma and has become the procedure of choice in many major centres. However, its efficacy at long-term follow-up compared to the open approach, particularly in neurologically impaired children, has not been investigated.

**What this study adds**

- This follow-up study of a randomised controlled trial indicates that laparoscopic Nissen fundoplication is associated with reduced incidence of retching at long-term follow-up, a finding not previously reported in children.

## Introduction

Nissen fundoplication is commonly performed for surgical treatment of gastro-oesophageal reflux (GER) in children. Paediatric surgeons who perform the Nissen fundoplication believe that laparoscopy provides more rapid postoperative recovery and early discharge from hospital. A study of 127 children who underwent laparoscopic Nissen fundoplication (LNF) has shown that this technique is effective in reducing the incidence of GER at 5.5 years follow-up.^1^ Recently Kubiak and colleagues reported a failure rate of 5.9% at long-term follow-up in children following LNF.^2^ Randomised controlled trials (RCTs) in adults show that the laparoscopic procedure is as effective as the open procedure in improving acid reflux at long-term follow-up.^3^ Nevertheless, although evidence from the literature suggests that the median time from initial fundoplication to the recurrence of GER in children is 1.5 years,^4^ there are no studies comparing the results after open (ONF) and LNF at long-term follow-up. A RCT on children assigned to receive ONF or LNF was performed in our Institution,^5^ with short-term (22 months) clinical outcomes reported.^6^ The aim of the present study was to perform a longer term follow-up (up to 5 years) to establish which group has the best results.

## Methods

This is a follow-up study of children randomised 4 years earlier to undergo ONF or LNF over a 2-year period. The primary aim of the original trial was to compare the resting energy expenditure (REE) between the two groups. Data from previous studies was used for the power calculation to detect a difference of 1 SD in the 4-h postoperative REE between the 2 groups; using a significance level of 5%, required 21 per group for 90% power.^5^ ^7^ Patients were randomised to ONF or LNF using the programme Minim (London Hospital Medical School). All children requiring Nissen fundoplication over the 2-year period were assessed for eligibility in the study. Exclusion criteria included major cardiac or renal anomalies and/or congenital metabolic abnormalities. Minimisation criteria included patient age, neurologic status and operating surgeon. Operations were performed by four paediatric surgeons with comparable experience in both procedures. The surgical techniques were standardised, as was the general anaesthesia. The ONF was performed via an upper midline incision. The liver was retracted superiorly and the phrenoesophageal ligament and gastrohepatic omentum divided exposing the oesophageal hiatus. The right and left crura were approximated using interrupted non-absorbable sutures. The short gastric vessels were not divided. A 360° wrap of the fundus was fashioned using interrupted non-absorbable sutures. The wrap was not sutured to the diaphragm. If necessary a Stamm gastrostomy was performed. The LNF was performed with three ports. A 5-mm Hasson cannula for the 30° telescope was placed above the umbilicus. Two ports for the instruments were placed in right and left upper quadrants. A Nathanson retractor (Cook Medical Europe Ltd.) was inserted in the epigastrium to retract the liver superiorly. The rest of the operation was performed as for the ONF. The procedure was converted to open in the presence of intraoperative anaesthetic or surgical complications (eg, profuse bleeding, organ injuries) deemed not amenable to be dealt laparoscopically. The postoperative feeding regime was standardised for both groups until the child was fully fed and discharged home. After discharge, the feeding regime was adapted to the child's and family's needs.

Patients included in this initial RCT have been under regular follow-up and were invited to participate in a second study to determine the long-term outcome. A separate ethical approval and written consent were obtained. The study was registered with a database of RCTs (*clinicaltrials.gov* identifier NCT00259961). The study was conducted by an independent investigator (MP) not involved in the initial trial or clinical management of the children and unaware of the procedure that the children were originally allocated. Recurrence of GER was investigated by upper gastrointestinal contrast study and/or by 24-h pH study in all symptomatic patients. For ethical reasons, asymptomatic children did not undergo postoperative investigations. Incidence of retching (unsuccessful effort to vomit), gas bloat syndrome (inability to belch and/or ‘degas’ the stomach) and dumping syndrome (presence of flushing, sweating, dizziness, weakness and vasomotor collapse after eating) was recorded. Nutritional status at surgery and follow-up was evaluated according to weight and body mass index (BMI) Z-scores calculated using British 1990 reference data.^8^ A questionnaire designed for neurologically impaired children and modified for neurologically normal children was used to assess the quality of life before surgery, 6 months after surgery and at follow-up.^9^ The questionnaire included three different parts: ‘Daily Care and the Overall Condition of The Child’; ‘Child and Parents’ Overall Quality of Life; ‘Child's Special Medical Needs’. Questions scored from 1 (best) to 5 (worst). Parents were asked to classify the results of surgery ‘better than expected’, ‘about as expected’ and ‘worse than expected’. Data are reported as median (range), with IQR, and were compared by repeated measures ANOVA (Friedman test), Mann–Whitney U test, χ^2^ test and Fisher’s exact test using GraphPad Prism V.5.03 (GraphPad Software, Inc.).

## Results

**Figure 1:**
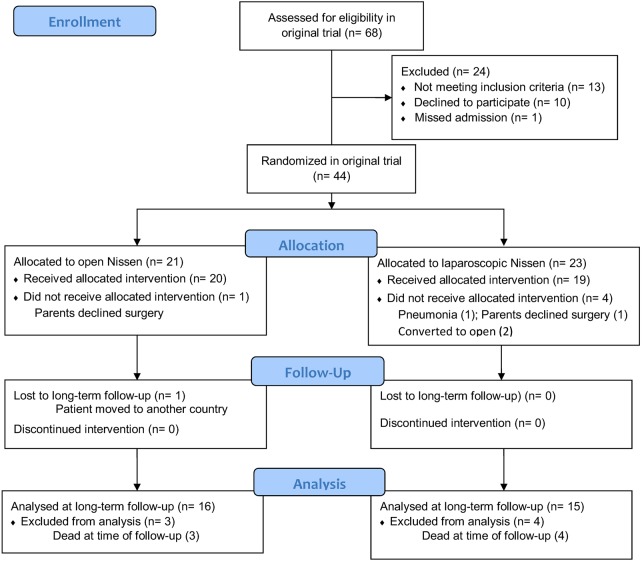
Flow diagram showing patients’ recruitment from original study to long-term follow-up.

Sixteen and 15 patients randomised in the original trial were available at follow-up in the open and laparoscopic group, respectively (figure 1). Two patients randomised to LNF were converted to ONF in the original trial: 1 patient had a significant respiratory deterioration before surgery and underwent ONF. The other patient was converted for difficulties in identifying the anatomy. These patients were excluded from the follow-up study as it was felt not appropriate to perform an intention-to-treat analysis as the aim of the study was to assess the effects of the actual operation performed.

The two groups were comparable with respect to age and weight at the time of operation, neurological status and surgeon performing the operation at the time of randomisation. Furthermore, there were no differences between the patients available at follow-up in the two groups (table 1).

**Table 1:** Demographics of patients available at follow-up

	Open	Laparoscopic	p Value
Available at follow-up	16	15	ns
Gender (M:F)	7:9	9:6	ns
Age at Nissen fundoplication (years)	4.3 (0.4–16.4)	7.5 (0.3–18.6)	ns
Weight at Nissen fundoplication (kg)	11.1 (4–37)	16.0 (4.7–43.4)	ns
Neurological impairment	12 (75%)	13 (86%)	ns
Gastrostomy insertion at time of Nissen fundoplication	8 (50%)	12 (80%)	ns
Age at follow-up (years)	8.6 (4.1–20.5)	11.3 (4.8–23.6)	ns
Length of follow-up (years)	4.1 (3.1–5.3)	4.1 (2.6–5.1)	ns

Follow-up was 4.1 years (3.1–5.3) for the ONF group and 4.1 years (2.6–5.1) for the LNF group (p=0.9). Seven neurologically impaired children (3 ONF, 4 LNF) died at the age of 3.6 years (0.6–10.1) after a median of 10 months (range 1.0–29.0) following surgery (table 2).

**Table 2:** Characteristics of 7 neurologically impaired children who had died between Nissen fundoplication and the beginning of the follow-up

	Type of Nissen	Diagnosis	Gender	Age at Nissen (years)	Time from Nissen to death (months)	Cause of death
Patient 1	ONF	Congenital myopathy	Male	0.5	2.9	Progressive respiratory failure
Patient 2	ONF	Cerebral palsy, microcephaly	Female	8.9	14.1	Varicella related pneumonia
Patient 3	ONF	Lesch-Nyhan syndrome	Male	8.0	23.1	Chronic renal failure
Patient 4	LNF	Ex-premature, Cerebral palsy, chronic lung disease, tracheostomy	Male	3.5	1.0	Progressive respiratory failure
Patient 5	LNF	Cerebral palsy	Female	1.7	10.0	Progressive encephalitis
Patient 6	LNF	Sphingolipidosis	Male	3.5	29.0	Chronic renal failure
Patient 7	LNF	Ex-premature, Congenital malformation of corpus callosum, epilepsy, chronic lung disease, tracheostomy	Female	0.3	4.2	Progressive respiratory failure

LNF, laparoscopic Nissen fundoplication; ONF, open Nissen fundoplication.

At time of death there was no evidence of recurrent GER in any of the patients and none of them was on antireflux medications. Although 4 patients died of respiratory problems, the causes were unrelated to recurrent aspiration of gastric content secondary to failed Nissen fundoplication. In details: patient 1 died of respiratory failure secondary to congenital myopathy; patient 2 died of pneumonia from varicella infection; patient 4 and 7 died of chronic lung disease related to extreme prematurity.

Incidence of recurrent reflux was similar for the two groups with 2 (12.5%) ONF patients and 3 (20%) LNF (p=ns) with documented recurrent gastro-oesophageal reflux (confirmed by upper G-I study in all and pH study in 2). None of the other patients had documented reflux or was receiving antireflux medication at follow-up. Four (80%) of the 5 patients with failure were neurologically impaired. One patient in each group required redo-Nissen fundoplication; the remaining 3 patients (1 ONF and 2 LNF) with recurrent GER were successfully managed with medications. Postoperative gastrointestinal symptoms are reported in table 3.

**Table 3:** Postoperative findings at follow-up in 31 surviving patients (16 in open group and 15 in laparoscopic group)

	Open	Laparoscopic	p Value
Retching	8 (50%)	1 (7%)	0.01
Gas bloat syndrome	5 (31%)	2 (13%)	ns
Dumping syndrome	1 (6%)	1 (6.5%)	ns
Any of the above	9 (56.2%)	4 (26.6%)	ns

Incidence of retching was significantly higher in the open group compared to the laparoscopic group (50% vs 7%, p=0.01) at long-term follow-up. All patients with retching at long-term follow-up were patients in whom retching was already present in the early postoperative period (reported by McHoney and colleagues^6^). However, 2 patients in the open group had retching in the early postoperative period but not at long-term follow-up.

**Figure 2:**
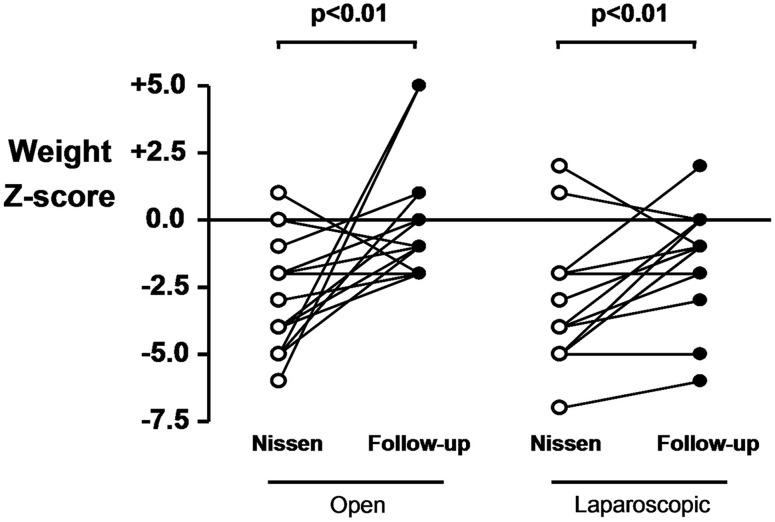
Weight Z-score in the two groups before and after surgery (follow-up).

**Figure 3:**
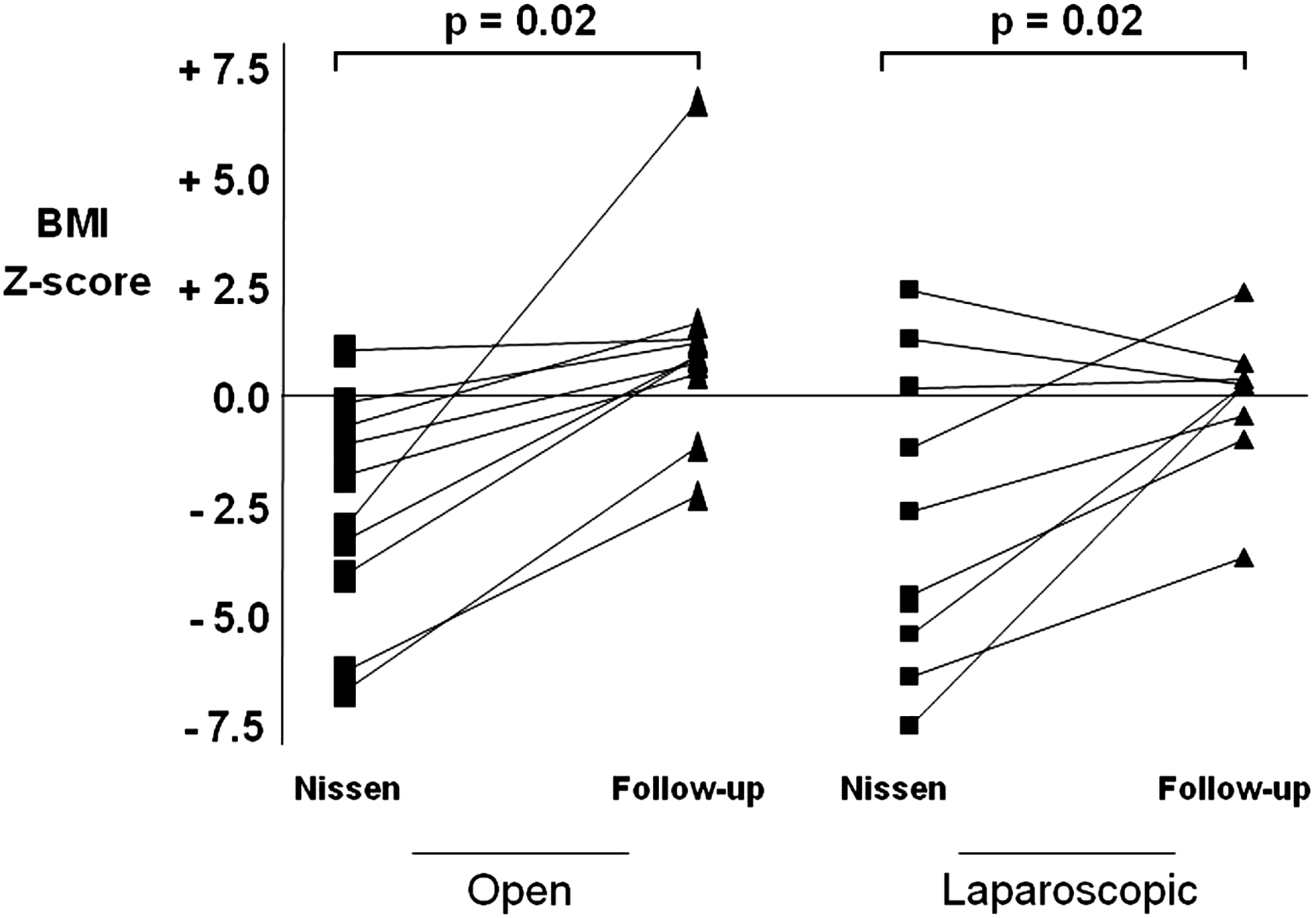
Body mass index (BMI) Z-score before and after surgery (follow-up).

Six (66.6%) patients out of the 9 with postoperative retching were neurologically impaired (5 patients in the ONF group and 1 patient in the LNF group). Six (75%) of the 8 patients in the ONF group and the patient in the LNF group with retching at follow-up had a gastrostomy. Nine (70%) patients out of the 13 with any symptoms were neurologically impaired (5 patients in the ONF group and 4 patients in the LNF group). Two (6.5%) neurologically impaired children (one in in each group) presented with late dumping syndrome. Nutritional status improved after surgery in both groups (excluding patients with recurrent reflux) as indicated by a significant increase in weight Z-score (p<0.01) (figure 2) and BMI Z-score (p=0.02 in ONF and p=0.01 in LNF) (figure 3). Quality of life at follow-up was significantly improved in both groups (p<0.001) with no differences between the two groups (tables 4–6).

**Table 4:** Daily care and the overall condition of the child (mean±SD) in the open (ONF) and laparoscopic (LNF) Nissen fundoplication group

	ONF	LNF
Ease of feeding		
6 months before surgery	4.3±0.7	4.1±1.0
6 months immediately postsurgery	2.6±0.9***	2.5±1.2**
At follow-up	2.2±0.9***	1.5±0.8***
Physical comfort during feeding		
6 months before surgery	3.9±1.3	4.1±1.0
6 months immediately postsurgery	2.4±1.1*	2.8±1.2*
At follow-up	2.3±1.3*	1.7±1.1***
Constipation		
6 months before surgery	1.7±1.3	2.4±1.6
6 months immediately postsurgery	1.8±1.3	2.4±1.6
At follow-up	1.6±1.3	2.2±1.5
Gas-bloat		
6 months before surgery	2.4±1.1	1.6±1.5
6 months immediately postsurgery	2.4±1.0	2.5±1.6
At follow-up	2.6±1.0	2.2±1.1
Pneumonias		
6 months before surgery	4.3±0.6	3.5±1.2
6 months immediately postsurgery	2.6±1.3**	2.1±0.9**
At follow-up	2.3±1.4***	2.2±1.2**
Comfort of child		
6 months before surgery	4.1±1.1	4.4±0.5
6 months immediately postsurgery	2.1±1.2***	2.1±0.7***
At follow-up	1.7±0.9***	1.7±1.0***
Child's ability to enjoy life		
6 months before surgery	4.2±1.1	4.4±0.5
6 months immediately postsurgery	2.7±1.1**	2.1±0.8***
At follow-up	1.9±1.1***	1.7±0.9***
Child's developmental progress		
6 months before surgery	3.6±1.2	3.8±0.9
6 months immediately postsurgery	2.6±1.0**	2.6±0.9*
At follow-up	1.9±0.9***	2.0±0.9***

Scale: 1, Excellent; 2, Good; 3, Average; 4, Poor; 5, Terrible.

*p<0.05; **p<0.01; ***p<0.001 vs 6 months before surgery.

**Table 5:** Child's and parental overall quality of life (mean±SD) in the open (ONF) and laparoscopic (LNF) Nissen fundoplication group

	ONF	LNF
Overall ease of caring for the child		
6 months before surgery	4.3±0.7	4.3±0.6
6 months immediately postsurgery	2.4±1.0***	2.0±0.8***
At follow-up	2.2±0.5***	1.9±1.2***
Overall enjoyment of the child		
6 months before surgery	3.9±0.9	4.0±1.1
6 months immediately postsurgery	2.1±1.1***	2.1±1.0***
At follow-up	1.6±0.7***	1.4±0.6***
Quality of time spent with the child		
6 months before surgery	4.1±1.1	4.1±0.7
6 months immediately postsurgery	1.2±1.3***	2.1±0.9***
At follow-up	1.5±0.7***	1.8±1.0***
Level of frustration		
6 months before surgery	3.6±1.1	3.9±1.2
6 months immediately postsurgery	2.4±1.1**	2.3±1.2**
At follow-up	1.7±0.7***	1.6±1.0***
Level of concern		
6 months before surgery	2.4±1.5	3.0±1.5
6 months immediately postsurgery	2.0±1.0	1.5±0.9**
At follow-up	1.1±0.3***	1.0±0.3***
Overall quality of life		
6 months before surgery	4.1±0.6	4.1±0.7
6 months immediately postsurgery	2.3±0.8***	1.9±1.0***
At follow-up	1.7±0.7***	1.5±0.6***

Scale: 1, Excellent; 2, Good; 3, Average; 4, Poor; 5, Terrible.

*p<0.05; **p<0.01; ***p<0.001 vs 6 months before surgery.

**Table 6:** Child's special medical needs (mean±SD) in the open (ONF) and laparoscopic (LNF) Nissen fundoplication group

	ONF	LNF
Time for medical/physical needs		
6 months before surgery	4.2±0.8	3.7±1.1
6 months immediately postsurgery	2.2±1.0***	2.2±0.4***
At follow-up	1.8±0.8***	1.8±0.6***
Visits to doctors and hospital		
6 months before surgery	4.4±0.7	3.9±1.2
6 months immediately postsurgery	2.3±1.0***	2.1±0.4***
At follow-up	2.1±0.8***	1.4±0.5***

Scale: 1, Excellent; 2, Good; 3, Average; 4, Poor; 5, Terrible.

*p<0.05; **p<0.01; ***p<0.001 vs 6 months before surgery.

A significant improvement was found in the quality of life at follow-up when compared to the preoperative period. Parents’ satisfaction was significantly high: 62% in the ONF and 73% in the LNF groups (p=ns) described the operation as ‘better than expected’, 38% and 27% respectively described the operation as ‘about as expected’ (p=ns). None of the family described the operation as ‘worse than expected’. There were no differences between the two groups.

## Discussion

This is the first long-term follow-up study of a RCT in children documenting that Nissen fundoplication improves the quality of life and controls GER independently of the technique. However, LNF is associated with a lower incidence of retching.

The ONF is associated with postoperative complications (up to 26%) including gas bloating, retching, vomiting, dumping syndrome and recurrence of reflux (up to 30%) particularly in neurologically impaired children.^4^ ^10^ ^11^ The LNF offers the advantage of decreasing the surgical trauma although its long-term efficacy has not been investigated. Evidence from the literature 4 suggests that the median time from fundoplication to the recurrence of GER is 1.5 years but most studies focus on the short term postoperative results. Tovar and colleagues have shown that LNF is as effective as the ONF in reducing the GER at short-term follow-up (19 months).^12^ Other authors have reported that LNF is a durable procedure in children after 3 and 5.5 years, respectively.^1^ ^13^ Furthermore, long-term results of RCTs in adults show that LNF is as effective as ONF in improving acid reflux.^3^ However, there are no similar RCTs in children. We previously completed a RCT on children assigned to receive ONF or LNF and reported on the perioperative and short-term (22 months) clinical outcomes.^6^ In the current study, we further investigated the results of the two techniques at a median follow-up of 4.1 years. The randomisation led to a similar distribution of neurologically impaired children in each arm of the trial and we minimised the bias associated with the researcher being part of the surgical team by having an independent surgeon performing the follow-up. We documented recurrent reflux in 16% of patients with similar incidence in the two groups and one patient in each group requiring redo-Nissen fundoplication with results comparable to large retrospective series.^14^ McHoney and colleagues documented a higher incidence of retching in the early postoperative period in the ONF group in our cohort of patients (10/18 (55.6%) in the ONF group vs 1/16 (6.3%) in the LNF group (p=0.02)).^6^ We now confirm that the incidence of postoperative retching is persistently higher after ONF compared to LNF at long-term follow-up. Only 2 patients out of 10 with early postoperative retching in the ONF were found not to have retching a long-term follow-up suggesting that the effects of the surgical procedure are lasting over a long-period of time. The incidence of gas bloat and dumping syndrome was similar in both groups. A RCT in adults found higher incidence of dysphagia for LNF leading to early conclusion of the study.^15^ However, in the recently published long-term results, the authors found no differences in postoperative dysphagia between the two groups.^16^ Other RCTs reported similar postoperative dysphagia incidence in both groups.^17–19^ It is likely that the mechanism leading to postoperative dysphagia in adults and retching in children is different. The incidence of retching in our population of ONF is 50% and comparable to recent published data.^21^ The incidence of retching after antireflux surgery in children reported in the literature is variable between 20% and 56%, higher in the neurologically impaired patients, and it has been documented in up to 63% of children requiring redo-fundoplication.^20^ ^21^ In our series 66.6% of children with postoperative retching were neurologically impaired with equal distribution in the two groups and the use of standardised surgical procedures makes any bias related to patient's selection unlikely. Retching following fundoplication in children might be related to handling of the stomach that could impair autonomic pathways associated with disturbed gastric electrical control activity and gastric dysrhythmia,^22^ alterations in gastric emptying^23^ and vagal nerve dysfunction.^24^ St Peter and colleagues recently demonstrated that minimal oesophageal mobilisation reduces the incidence of postoperative wrap migration and recurrent reflux.^25^ Although in our study the two techniques were standardised, during LNF the dissection of the phreno-oesophageal membrane and manipulation of oesophagus and stomach are likely to be less extensive possibly reducing the incidence of vagal nerve dysfunction and retching by preserving the gastric physiology. Further prospective studies are warranted to verify this theory and clarify the gastric physiology following antireflux surgery. Postoperative retching could be also related to wrap herniation. However, the finding of higher incidence of retching in the open group was already evident in the immediate postoperative period as reported by McHoney and colleagues.^6^ In this respect, it is unlikely that the retching is related to herniation of the wrap. In addition, although children with retching did not have a contrast study to exclude wrap herniation, none of them presented with symptoms of recurrent reflux making the presence of wrap herniation unlikely. One limitation of our study is that there were only 16 patients after ONF and 15 patients after LNF alive at follow-up which could not allow detecting differences in incidence of recurrent reflux. However, the study demonstrates that the reduction in the incidence of retching in the laparoscopic group is significant even at 4-year follow-up. Hence, the laparoscopic technique may offer an advantage as it has been documented that the presence of severe postoperative retching is associated with a high recurrence rate of GER.^21^

Nutritional status was improved in both groups, as indicated by a significant increase in weight Z-score (p<0.001) and BMI Z-score (p=0.02 in ONF and p=0.01 in LNF). Regarding the quality of life, few studies have focused on parental satisfaction after antireflux surgery in children.^9^ ^26^ ^27^ Srivastava and colleagues documented that quality of life in children with neurological impairment is improved 1 month after surgery.^26^ Similarly, we found that Nissen fundoplication (open or laparoscopic) significantly improves the ease of care and reduces the child's medical needs improving the overall quality of life of the family. The majority of parents were satisfied with the results after the Nissen fundoplication and better quality of life based on caregivers evaluation was found in neurologically impaired and normal children. Improvement from severe vomiting is the likely explanation for the positive outcome of surgery. Nonetheless, the improved ease of feeding and physical comfort during feeding, together with an improved ability of the child to enjoy life might have contributed to better overall quality of life. In this respect, the gastrostomy was performed in more than 50% of children and might have influenced the child's condition by improving the ease of feeding; prior to surgery children were mainly fed by nasogastric tube that has disadvantages (frequent dislodgment, nose bleeding and skin excoriation). Also, in patients with gastrostomy, the opening of the tube leads to decompression of the stomach with reduction of the discomfort caused by the gas bloat. The reduction of the time spent for the child’s needs and the decreased numbers of physician/hospital visits had a significant effect in the parents’ satisfaction and this is particularly important since it can lead to improved work productivity and reduced economic costs for the family.

In conclusion, this is the first follow-up study of a RCT in children requiring Nissen fundoplication. Open and laparoscopic operations provide similar control of the GER after 4 years of follow-up leading to an improvement of the quality of life of the family. Furthermore, this study indicates that LNF is associated with reduced incidence of retching persisting at long-term follow-up, a finding not previously reported in children.

## References

[1] Capito C Leclair MD Piloquet H Long-term outcome of laparoscopic Nissen-Rossetti fundoplication for neurologically impaired and normal children. Surg Endosc 2008;22:875–80 17963001 10.1007/s00464-007-9603-3

[2] Kubiak R Andrews J Grant HW Long-term outcome of laparoscopic Nissen fundoplication compared with laparoscopic that fundoplication in children: a prospective, randomized study. Ann Surg 2011;253:44–9 21233605 10.1097/SLA.0b013e3181fc98a0

[3] Salminen PT Hiekkanen HI Rantala AP Comparison of long-term outcome of laparoscopic and conventional Nissen fundoplication: a prospective randomized study with an 11-year follow-up. Ann Surg 2007;246:201–6 17667497 10.1097/01.sla.0000263508.53334.af PMC1933575

[4] Kimber C Kiely EM Spitz L The failure rate of surgery for gastro-oesophageal reflux. J Pediatr Surg 1998;33:64–6 9473102 10.1016/s0022-3468(98)90363-3

[5] McHoney M Eaton S Wade A Inflammatory response in children after laparoscopic vs open Nissen fundoplication: randomized controlled trial. J Pediatr Surg 2005;40:908–13 15991169 10.1016/j.jpedsurg.2005.03.003

[6] McHoney M Wade AM Eaton S Clinical outcome of a randomized controlled blinded trial of open versus laparoscopic Nissen fundoplication in infants and children. Ann Surg 2011;254:209–16 21725231 10.1097/SLA.0b013e318226727f

[7] McHoney M Eaton S Wade A Effect of laparoscopy and laparotomy on energy and protein metabolism in children: a randomized controlled trial. J Pediatr 2010;157:439–44, 444 20400097 10.1016/j.jpeds.2010.02.067

[8] Freeman JV Cole TJ Chinn S Cross sectional stature and weight reference curves for the UK, 1990. Arch Dis Child 1995;73:17–24 7639543 10.1136/adc.73.1.17 PMC1511167

[9] O'Neill JK O'Neill PJ Goth-Owens T Care-giver evaluation of anti-gastroesophageal reflux procedures in neurologically impaired children: what is the real-life outcome? J Pediatr Surg 1996;31:375–80 8708906 10.1016/s0022-3468(96)90741-1

[10] Pearl RH Robie DK Ein SH Complications of gastroesophageal antireflux surgery in neurologically impaired versus neurologically normal children. J Pediatr Surg 1990;25:1169–73 2273433 10.1016/0022-3468(90)90756-y

[11] Spitz L Roth K Kiely EM Operation for gastro-oesophageal reflux associated with severe mental retardation. Arch Dis Child 1993;68:347–51 8466236 10.1136/adc.68.3.347 PMC1793892

[12] Tovar JA Olivares P Diaz M Functional results of laparoscopic fundoplication in children. J Pediatr Gastroenterol Nutr 1998;26:429–31 9552139 10.1097/00005176-199804000-00012

[13] Bourne MC Wheeldon C MacKinlay GA Laparoscopic Nissen fundoplication in children: 2–5-year follow-up. Pediatr Surg Int 2003;19:537–9 13680291 10.1007/s00383-003-0985-6

[14] Fonkalsrud EW Ashcraft KW Coran AG Surgical treatment of gastroesophageal reflux in children: a combined hospital study of 7467 patients. Pediatrics 1998;101:419–22 9481007 10.1542/peds.101.3.419

[15] Bais JE Bartelsman JF Bonjer HJ Laparoscopic or conventional Nissen fundoplication for gastro-oesophageal reflux disease: randomised clinical trial. The Netherlands Antireflux Surgery Study Group. Lancet 2000;355:170–4 10675115 10.1016/s0140-6736(99)03097-4

[16] Draaisma WA Rijnhart-De Jong HG Broeders IA Five-year subjective and objective results of laparoscopic and conventional Nissen fundoplication: a randomized trial. Ann Surg 2006;244:34–41 16794387 10.1097/01.sla.0000217667.55939.64 PMC1570591

[17] Ackroyd R Watson DI Majeed AW Randomized clinical trial of laparoscopic versus open fundoplication for gastro-oesophageal reflux disease. Br J Surg 2004;91:975–82 15286957 10.1002/bjs.4574

[18] Chrysos E Tsiaoussis J Athanasakis E Laparoscopic vs open approach for Nissen fundoplication. A comparative study. Surg Endosc 2002;16:1679–84 11984689 10.1007/s00464-001-9101-y

[19] Nilsson G Larsson S Johnsson F Randomized clinical trial of laparoscopic versus open fundoplication: blind evaluation of recovery and discharge period. Br J Surg 2000;87:873–8 10931021 10.1046/j.1365-2168.2000.01471.x

[20] Jolley SG Tunell WP Leonard JC Gastric emptying in children with gastroesophageal reflux. II. The relationship to retching symptoms following antireflux surgery. J Pediatr Surg 1987;22:927–30 3681624 10.1016/s0022-3468(87)80591-2

[21] Baerg J Thorpe D Bultron G A multicenter study of the incidence and factors associated with redo Nissen fundoplication in children. J Pediatr Surg 2013;48:1306–11 23845623 10.1016/j.jpedsurg.2013.03.028

[22] Richards CA Andrews PL Spitz L Nissen fundoplication may induce gastric myoelectrical disturbance in children. J Pediatr Surg 1998;33:1801–5 9869055 10.1016/s0022-3468(98)90289-5

[23] Jolley SG Tunell WP Leonard JC Gastric emptying in children with gastroesophageal reflux. II. The relationship to retching symptoms following antireflux surgery. J Pediatr Surg 1987;22:927–30 3681624 10.1016/s0022-3468(87)80591-2

[24] Lindeboom MY Ringers J van Rijn PJ Gastric emptying and vagus nerve function after laparoscopic partial fundoplication. Ann Surg 2004;240:785–90 15492559 10.1097/01.sla.0000143124.30911.0f PMC1356483

[25] St Peter SD Barnhart DC Ostlie DJ Minimal vs extensive esophageal mobilization during laparoscopic fundoplication: a prospective randomized trial. J Pediatr Surg 2011;46:163–8 21238659 10.1016/j.jpedsurg.2010.09.081 PMC3097032

[26] Srivastava R Downey EC Feola P Quality of life of children with neurological impairment who receive a fundoplication for gastroesophageal reflux disease. J Hosp Med 2007;2:165–73 17549766 10.1002/jhm.167

[27] Engelmann C Gritsa S Gratz KF Laparoscopic anterior hemifundoplication improves key symptoms without impact on GE in children with and children without neurodevelopmental delays. J Pediatr Gastroenterol Nutr 2010;51:437–42 20531026 10.1097/MPG.0b013e3181d1f1c8

